# Synthesis of P(AM/AA/SSS/DMAAC-16) and Studying Its Performance as a Fracturing Thickener in Oilfields

**DOI:** 10.3390/polym17020217

**Published:** 2025-01-16

**Authors:** Shuai Wang, Lanbing Wu, Lu Zhang, Yaui Zhao, Le Qu, Yongfei Li, Shanjian Li, Gang Chen

**Affiliations:** 1 No. 4 Gas Production Plant of Yanchang Gas Field, Shaanxi Yanchang Petroleum (Group) Company Limited, Yan’an 716000, China; wangshuai@sxycpc.com; 2 Engineering Research Center of Oil and Gas Field Chemistry, Universities of Shaanxi Provence, Xi’an Shiyou University, Xi’an 710065, China; wulanbing@sinochem.com (L.W.); qule@xsyu.edu.cn (L.Q.); yfli@xsyu.edu.cn (Y.L.); 3 Shaanxi Sinochem Lantian New Materials Co., Ltd., Weinan 710000, China; 4 No. 11 Oil Production Plant, Changqing Oilfield Company, Qinyang 745000, China; lzhw_cq@petrochina.com.cn (L.Z.); zhaoyarui_cq@petrochina.com.cn (Y.Z.)

**Keywords:** reversed-phase emulsion polymerization, emulsion fracturing fluid thickener, instant thickening

## Abstract

In order to solve the problems of long dissolution and preparation time, cumbersome preparation, and easy moisture absorption and deterioration during storage or transportation, acrylamide (AM), acrylic acid (AA), sodium p-styrene sulfonate (SSS), and cetyl dimethylallyl ammonium chloride (DMAAC-16) were selected as raw materials, and the emulsion thickener P(AM/AA/SSS), which can be instantly dissolved in water and rapidly thickened, was prepared by the reversed-phase emulsion polymerization method. DMAAC-16, the influence of emulsifier dosage, oil–water ratio, monomer molar ratio, monomer dosage, aqueous pH, initiator dosage, reaction temperature, reaction time, and other factors on the experiment was explored by a single-factor experiment, and the optimal process was determined as follows: the oil–water volume ratio was 0.4, the emulsifier dosage was 7% of the oil phase mass, the initiator dosage was 0.03% of the total mass of the reaction system, the reaction time was 4 h, the reaction temperature was 50 °C, the aqueous pH was 6.5, and the monomer dosage was 30% of the total mass of the reaction system (monomeric molar ratio n(AM):n(AA):n(SSS):n(DMAAC-16) = 79.2:20:0.5:0.3). X-ray diffraction analysis (XRD), infrared spectroscopy (FTIR), thermogravimetric analysis (TGA), and scanning electron microscopy analysis were carried out on the polymerization products. At the same time, a series of performance test experiments such as thickening performance, temperature and shear resistance, salt resistance, sand suspension performance, core damage performance, and fracturing fluid flowback fluid reuse were carried out to evaluate the comprehensive effect and efficiency of the synthetic products, and the results show that the P(AM/AA/SSS/DMAAC-16) polymer had excellent solubility and excellent properties such as temperature and shear resistance.

## 1. Introduction

With the rapid development of China’s oil and gas industry, oil and gas resources are gradually degrading, and fracturing is the most important production increase measure for oil and gas development. When fracturing is carried out, the performance of the fracturing fluid is a key factor affecting the effect of reservoir modification [1,2]. As the core component of the fracturing system, the thickener in the fracturing fluid plays an important role in adjusting viscosity, improving fluidity, controlling fracture size, and improving sand overhang. At present, the common thickeners at home and abroad are mainly divided into three categories: vegetable gums represented by guar gum, surfactants represented by viscoelastic surfactants (VESs), and polymers represented by polyacrylamide. However, the fracturing fluid system prepared by these thickeners has a high residue content and great damage to the reservoir and is greatly affected by the market demand for guar gum as well as the weather and other growth factors, which greatly limits their use in oilfield fracturing operations [3,4]. Viscoelastic surfactant thickeners have poor temperature resistance and high production costs, making them difficult to scale up [5,6]. The fracturing fluid system prepared by polymeric thickener has become a hot spot due to its advantages of low friction, low residue content, and low damage to the reservoir. Ge Y et al. [7] used AM, AA, AMPS, and cetyl dimethylammonium bromide (DM16) as raw materials to prepare a betaine hydrophobic association polymer JDL-1 thickener by mixed micellar aqueous solution polymerization using a redox initiation system. When the surfactant was used for physical cross-linking, the apparent viscosity of the cross-linked fracturing fluid remained above 60 mPa·s after 1 h of shearing under the experimental conditions of 90 °C and 170 s^−1^. Ma X et al. [8] synthesized the two-tailed hydrophobic monomer PMB-16 with AM and methacryloyloxyethyltrimon ammonium chloride (DMC) by radical copolymerization to synthesize a ternary hydrophobic association polymer (PADM). The performance evaluation results show that the PADM aqueous solution has excellent high-temperature resistance and salt resistance. Yan S et al. [9] prepared a novel hydrophobic association temperature-resistant thickener LK by free radical copolymerization using AM, AA, sodium p-styrene sulfonate (SSS), and dimethyl diallyl ammonium chloride as raw materials. The experimental results show that the fracturing gel system has good comprehensive properties under high-temperature conditions. However, the powder polymer fracturing fluid thickener will undergo an obvious swelling–dissolution process during the preparation process. The polymer molecular chain is completely stretched, and it takes a certain time for the solution viscosity to reach the maximum value, which made it difficult to meet the needs of online continuous mixing. Therefore, in this paper, acrylamide (AM), acrylic acid (AA), sodium ter-styrene sulfonate (SSS), and cetyl dimethylallyl ammonium chloride (DMAAC-16) were used as raw materials to prepare an emulsion-type thickener that can be instantly dissolved in water and rapidly thickened by reversed-phase emulsion polymerization, and the preparation process of the thickener was explored. Then, a one-dose fracturing fluid thickener, P(AM/AA/SSS/DMAAC-16), with excellent solubility and temperature and shear resistance properties was developed.

## 2. Materials and Methods

### 2.1. Experimental Materials

Acrylamide (AR) was purchased from Tianjin Damao Chemical Reagent Factory (North Yubao Industrial Zone, Huaming Street, Dongli District, Tianjin, China); acrylic acid (AR) was purchased from Tianjin Damao Chemical Reagent Factory; sodium p-styrene sulfonate (AR) was purchased from Shanghai Darui Fine Chemicals Co., Ltd., (Room 2049, Zone O, 2nd Floor, Building 1, No. 15, Lane 588, Liantang Road, Zhang Liantang Town, Qingpu District, Shanghai, China); cetyl dimethylallyl ammonium chloride (AR) was purchased from Zhangjiagang Hambadu Chemical Co., Ltd., (No.8, Xi Jia Road, Fenghuang, Fenghuang Town, Zhangjiagang City, China); Siban-80 (AR) was purchased from Tianjin Damao Chemical Reagent Factory; Tween-60 (AR) was purchased from Tianjin Damao Chemical Reagent Factory; 5# white oil (AR) was purchased from Tiancheng Meijia Lubricants (Beijing) Co., Ltd., (Room 2-6113, Block A, No. 6 Huafeng Road, Huaming Hi-Tech Industrial Zone, Dongli District, Tianjin, China); Emulsifier OP-10 (AR) was purchased from Tianjin Fuyu Fine Chemical Co., Ltd., (North Road East, Beixiaoliang Village, Daliang Town, Wuqing District, Tianjin, China); V50 (AR) was purchased from Nanjing Dulai Biotechnology Co., Ltd., (Room 503, Building 2, Chuangxinhui, No. 61 Wenjing Road, Jiangbei New District, Nanjing, China); and sodium hydroxide (AR) was purchased from Tianjin Damao Chemical Reagent Factory.

### 2.2. Preparation of Thickener

**Figure 1:**
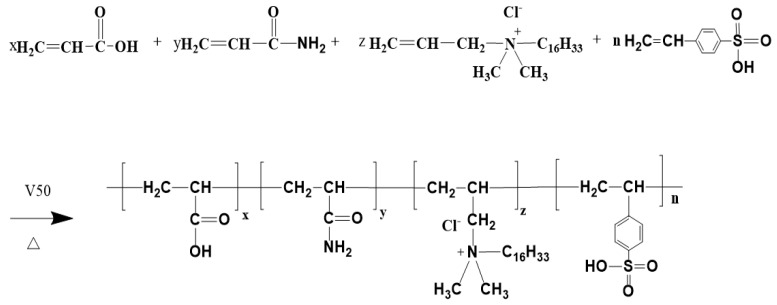
P(AM/AA/SSS/DMAAC-16) reaction.

For the selection of monomers, AM, which has good polymerization performance, high reactivity and excellent water solubility, was chosen as the main chain structure of linear water-soluble molecules, and quaternary ammonium salts containing sixteen carbons of long-chain alkyl cationic hydrophobic monomers, DMAAC-16, and SSS with benzene ring structure were introduced sequentially, with the former used to improve polymer binding and temperature resistance, and the latter used to improve the rigidity of polymer molecular chains, as well as the salt resistance. and salt resistance. According to the mass ratio of 9:1, a certain amount of Span-80 and Tween-60 was mixed in 5# white oil to prepare the oil phase. Then, an appropriate amount of monomers AM, AA, SSS, and DMAAC-16 was mixed and dissolved in distilled water in a certain proportion to prepare the aqueous phase, and the pH of the aqueous phase was adjusted to 6~7. The oil phase was transferred to a three-neck flask with a constant-pressure dropping funnel, a thermometer, and a nitrogen connection device, and the aqueous phase was dropped into the oil phase by the constant-pressure dropping funnel for 25 min under the action of magnetic stirring at a speed of 400 r/min; the water phase was pre-emulsified for 25 min to obtain a water-in-oil fine emulsion with high stability. After the pre-emulsification, the prepared V50 solution was added to the constant-pressure dropping funnel in advance, and then the nitrogen combination device was opened to continuously deoxygenate the three-neck flask for 40 min to ensure that the polymerization reaction was carried out in an oxygen-free environment. After 40 min, the water bath temperature of the collector thermostatic heating magnetic stirrer was adjusted to 50 °C, and the change in the thermometer in the emulsion system was observed; when the temperature of the thermometer rose to 50 °C, the constant-pressure dropping funnel was opened, and the initiator V50 solution was slowly added dropwise to initiate the polymerization reaction. At the same time, the heating rate of the emulsion system was maintained at 0.3–0.5 °C/min by adjusting the flow rate and dosage of the initiator (if the heating rate is too fast, ice water bath cooling can be considered), so that the polymerization reaction can be carried out smoothly for 3–4 h. When the temperature of the initiator system no longer changes, the polymerization reaction is completed. When the reaction product has cooled to room temperature, an appropriate amount of phase converter with a large hydrophilic and lipophilic balance value (HLB) is added to obtain an emulsion-type fracturing fluid thickener that can be instantly dissolved in water. The polymerization reaction formula of the thickener is shown in Figure 1.

### 2.3. Apparent Viscosity Determination

The main technical index of this experiment is the determination of the apparent viscosity of the solution. A six-speed rotational viscometer is used, and the measured viscosity is dynamic viscosity. The apparent viscosity of the solution is tested according to the standard [10] General Technical Requirements for Vegetable Gum for Fracturing.

(1) Preparation

First, 350 mL of distilled water was added to the container, then 3.5 g of fracturing fluid thickener P (AM/AA/SSS/DMAAC-16) was slowly added under the condition of continuous low-speed stirring, and the fracturing fluid solution with a concentration of 1% was obtained by stirring fully and was then poured into a beaker.

(2) Data measurement

The speed of the six-speed rotational viscometer was adjusted to 100 r/min, and the viscosity of the liquid at this speed was tested. The reading value was read and recorded when the red pointer on the six-speed rotational viscometer indicator plate was stable.

(3) Data Processing

The apparent viscosity of the gel is calculated according to Equation (1):

(1) $$\mu =\frac{5.007\;\alpha }{1.704}$$

The meaning of each parameter is as follows: *μ*—apparent viscosity value, mPa·s; *α*—rotational viscometer dial reading; 5.077—*α* is 1 at this shear stress, the unit is 10^−1^ Pa; 1.704—the corresponding shear rate at the same speed, s^−1^.

### 2.4. Determination of Thickening Performance

The thickener P(AM/AA/SSS/DMAAC-16) was prepared into a 1% solution with water, and the operation method refers to 1.3. A six-speed viscometer was used to test the apparent viscosity of the solution at 20 s intervals. Finally, the relationship between the apparent viscosity of the polymer solution and the change in time was obtained.

### 2.5. Determination of Temperature Resistance

The thickener P(AM/AA/SSS/DMAAC-16) solution was prepared; refer to 1.3 for the operation method. The temperature resistance of the thickener solution was tested by using the MARS60 (Haaker, Germany) complex fluid flow tester, and finally the relationship between the apparent viscosity of the polymer solution and the temperature was obtained.

### 2.6. Determination of Shear Resistance

The thickener P(AM/AA/SSS/DMAAC-16) gel was prepared; refer to 1.3 for the operation method. The shear resistance of the thickener solution was tested by using the MARS60 complex fluid flow tester, and finally the relationship between the apparent viscosity of the polymer solution and the shear time was obtained.

### 2.7. Determination of Salt Tolerance

NaCl solutions and CaCl_2_ solutions with different gradient salinities (0, 5000, 10,000, 15,000, and 20,000 ppm) were prepared in simulated water. The thickener P(AM/AA/SSS/DMAAC-16) was prepared into a 1% solution with the above simulated water, and for the operation method, we refer to 1.3. The apparent viscosity of the polymer solution was measured by a six-speed viscometer, and finally the relationship between the apparent viscosity of the polymer solution and the salinity of the simulated water was obtained.

### 2.8. Viscoelastic Performance Determination

The thickener P(AM/AA/SSS/DMAAC-16) was prepared into a 1% solution with water, and the operation method refers to 1.3. The viscoelastic performance of the thickener solution was tested by the MARS60 complex fluid flow tester, and the stress sweep and frequency sweep of the two thickener solutions were carried out, respectively. The stress scanning test conditions were as follows: the test temperature was 25 °C, the scanning frequency was 1 Hz, and the scanning range was 0.01–100 Mpa. The frequency sweep test conditions were as follows: the test temperature was 25 °C, the shear stress was 1 Pa, and the frequency sweep range was 0.1–10 Hz.

### 2.9. Determination of Sand Suspension Performance

The thickener P(AM/AA/SSS/DMAAC-16) was prepared into a 1% solution with water, and the operation method refers to 1.3. At room temperature and 90 °C, 20/40 ceramsite was selected as proppant to test the sand-carrying capacity of the thickener solution after adding 20%, 30%, 50%, and 100% sand ratios to 1% thickener solution according to the People’s Republic of China oil and gas industry standard [11] (SY/T 5185-2016).

### 2.10. Determination of Gel Breaking Performance

The thickener P (AM/AA/SSS/DMAAC-16) was prepared into a 1% solution with water, and the operation method refers to 1.3. Ammonium persulfate, a 0.04% gel breaker, was added to the thickener solution and broken in a water bath at 90 °C. The surface tension and residue content of the broken gel solution were tested according to the People’s Republic of China oil and gas industry standard [12] (SY/T 5107-2016).

### 2.11. Determination of Core Damage Performance

The damage of 1% fracturing fluid thickener to the core was studied according to the oil and gas industry standard of the People’s Republic of China (SY/T 5107-2016) by using the water shutoff profile control evaluation instrument (DQ-IV type) of Jiangsu Huaan Scientific Research Instrument Co., Ltd., Nantong, China.

### 2.12. Characterization of Thickeners

The crystallization properties of the polymer were studied by using a D8 ADVAHCL X-ray(Bruker Technologies GmbH, Germany (Bruker) polycrystalline diffractometer with 40 kV and 30 mA working conditions under a Cu-Kα ray source (wavelength λ = 1.5406 Å), and 2θ was controlled in the range of 10° to 70°. The type and molecular structure of polymers were studied by infrared scanning with a Nicolet 5700 (Thermo Fisher Scientific, Waltham, MA, USA) infrared spectrometer in the range of 500–4000 cm^−1^. The thermal stability of the polymer was studied by a TGA thermogravimetric analyzer under the experimental conditions of a heating rate of 10 °C/min, a temperature space of 30~600 °C, atmosphere gas of N^2^, and a flow rate of 40.0 mL/min.

## 3. Results and Discussion

### 3.1. Determination of the Optimal Synthesis Conditions of Thickeners

#### 3.1.1. Effect of Emulsifier Dosage on Polymerization Reaction

**Figure 2:**
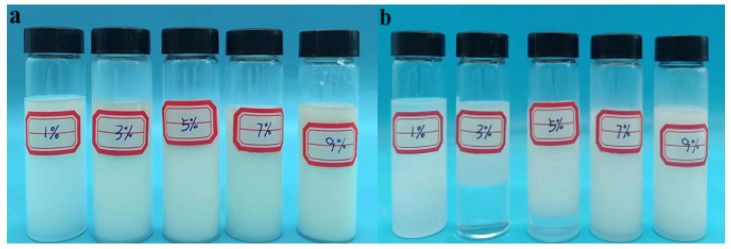
Effect of emulsifier dosage on reversed-phase emulsion system P(AM/SS/SSS/DMAAC-16). (**a**) The state before standing for 3d (**b**) The state after standing for 3d.

**Figure 3:**
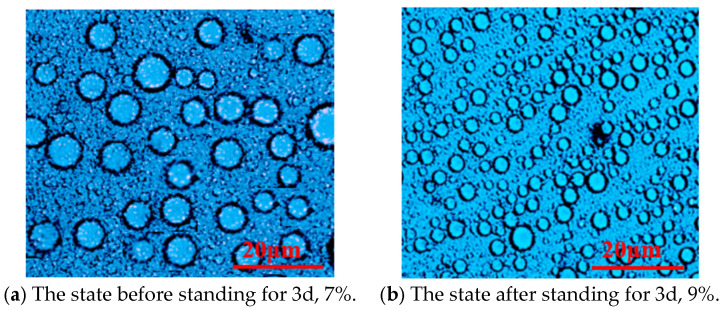
Microstructure of the emulsion system P(AM/SS/SSS/DMAAC-16) under different emulsifier contents.

Span-80 and Tween-60 composite emulsifiers (mass ratio of 9:1) were studied under the conditions of n(AM):n(AA):n(SSS):n(DMAAC-16) = 79.2:20:0.5:0.3; the oil–water volume ratio was 0.4, the monomer dosage accounted for 30% of the total mass of the reaction system, and the aqueous pH was 6.5. A series of P(AM/AA/SSS/C16-DMAAC) emulsions were synthesized from the different contents of the compound emulsifier Span-80/Tween-60 in the oil phase and allowed to stand for 3 days. The effect of emulsifier dosage on the stability of the reversed-phase emulsion system is shown in Figure 2 and Figure 3.

As can be seen from Figure 2, there is a positive correlation between the stability of the emulsion system and the amount of emulsifier. When the amount of emulsifier is too small, the reaction solution has a poor degree of pre-emulsification, and cannot even form a water-in-oil emulsion. When the compound emulsifier Span-80/Tween-60 accounted for 7% of the mass of the oil phase, the water-in-oil emulsion began to stabilize. As can be seen from Figure 3, the microdroplet size in the emulsion is optimal when the composite emulsifier Span-80/Tween-60 accounts for 7% of the oil phase mass. If the amount of emulsifier continues to increase, it is easy to form a fine emulsion with a small particle size. The small particle size of the droplets and the excessive number of polymerization sites make the content of reactant monomers in each site too small, which is not conducive to the chain growth of the polymer, resulting in a low molecular weight of the final synthesized polymer, which has an impact on the viscosity of the polymer solution. In summary, 7% is the optimal dosage of compound emulsifier for quaternary reversed-phase emulsion system.

#### 3.1.2. Effect of Oil–Water Ratio on Polymerization Reaction

**Figure 4:**
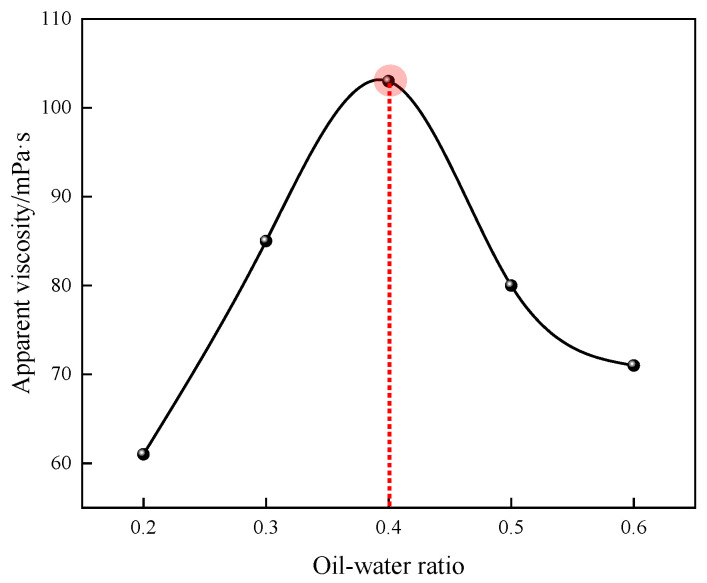
Effect of the oil–water ratio on the apparent viscosity of P(AM/SS/SSS/DMAAC-16) gel.

The effects of different oil–water ratios on the apparent viscosity of 1% quaternary polymer thickener solution were studied under the conditions of n(AM):n(AA):n(SSS):n(DMAAC-16) = 79.2:20:0.5:0.3; the amount of composite emulsifier accounted for 7% of the mass of the oil phase, the amount of monomer accounted for 30% of the total mass of the reaction system, the pH of the aqueous phase was 6.5, the amount of initiator accounted for 0.03% of the total mass of the reaction system, the reaction temperature was 50 °C, and the reaction time was 4 h. The results of the study are shown in Figure 4. As can be seen, the apparent viscosity of polymer P(AM/AA/SSS/DMAAC-16) gel increases first and then decreases with the gradual increase in the oil–water ratio. This is because when the ratio of oil to water is too low, the emulsion formed in the pre-emulsification stage is easy to stratify. Moreover, the excessive proportion of the aqueous phase directly leads to the extremely unstable reaction process, and the too small proportion of the oil phase directly leads to the heat of the polymerization reaction being difficult to dissipate, and finally, it is easy to cause the phenomenon of reaction overaggregation; the molecular weight of the final product obtained by the reaction is small, and the apparent viscosity of the polymer solution is low. With the gradual increase in the oil–water ratio, the oil phase can better disperse the molecules in the monomer droplets, and the reaction heat energy of the polymerization system is easier to disperse, so that the polymerization reaction chain grows smoothly, and the apparent viscosity of the polymer solution gradually increases. When the oil–water volume ratio was 0.4, the apparent viscosity of the 1% polymer solution was observed to be the largest, which was 103 mPa·s. However, when the proportion of oil and water continues to increase, the excessive proportion of the oil phase directly leads to the small particle size of the water-in-oil microemulsion produced in the pre-emulsification stage, which leads to there being too many polymerization sites, and the reaction monomer content in each site is too small, which ultimately leads to a small molecular weight of the product and a decrease in the apparent viscosity of the polymer solution. In summary, 0.4 is the optimal oil–water ratio for the quaternary polymerization reaction system.

#### 3.1.3. Effect of Monomer Molar Ratio on Polymerization Reaction

The effects of different monomer molar ratios on the apparent viscosity of 1% quaternary polymer thickener solution were studied under the conditions of an oil–water volume ratio of 0.4, an emulsifier dosage that accounted for 7% of the oil phase mass, a monomer dosage that accounted for 30% of the total mass of the reaction system, an aqueous phase pH of 6.5, an initiator dosage that accounted for 0.03% of the total mass of the reaction system, a reaction temperature of 50 °C, and a reaction time of 4 h. The results of the study are shown in Table 1.

Through the above experiments, it can be seen that when the content of hydrophobic monomers in the reaction system gradually increases, the intermolecular association of polymers in the solution is gradually enhanced, more cross-linking and interlaced structures between polymer molecular chains appear, and a large number of spatial network structures are continuously generated, so that the polymer molecules need to overcome greater resistance when flowing, and the viscosity of the polymer solution continues to increase. When the molar addition of DMAAC-16 was 0.3%, the maximum apparent viscosity of the 1% polymer solution was observed at 109 mPa·s. When the content of hydrophobic monomers continues to increase, a large steric hindrance will occur under the action of the hydrophobic long chain, the polymerization reaction will slow down, and the expansion and movement of polymer molecules will be limited, which will eventually lead to a decrease in the molecular weight of the polymer and the apparent viscosity of the polymer solution. At the same time, the apparent viscosity of the polymer thickener P(AM/AA/SSS/DMAAC-16) gel also increased first and then decreased with the addition of sodium styrene sulfonate (SSS), a functional monomer, because SSS was an anionic functional monomer and DMAAC-16 was a cationic functional monomer. When SSS increases in a certain range, it can improve the rigidity of the polymer molecular backbone, and once the range value is exceeded, the anionic and cationic monomers interact with each other to reduce the effective monomer content in the polymerization system, resulting in a decrease in the apparent viscosity of the polymer solution. Therefore, n(AM):n(AA):n(SSS):n(DMAAC-16) = 79.2:20:0.5:0.3 is the optimal monomer molar ratio for the quaternary polymerization reaction system

#### 3.1.4. Effect of Monomer Dosage on Polymerization Reaction

**Figure 5:**
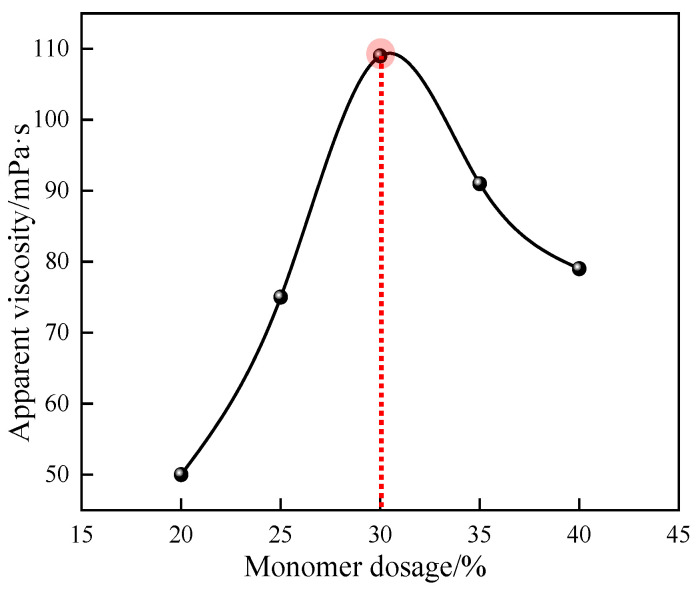
Effect of monomer dosage on apparent viscosity of P(AM/SS/SSS/DMAAC-16) gel.

The effects of different monomer dosage ratios on the apparent viscosity of 1% quaternary polymer thickener solution were studied under the conditions of n(AM):n(AA):n(SSS):n(DMAAC-16) = 79.2:20:0.5:0.3, an oil–water volume ratio of 0.4, an emulsifier dosage accounting for 7% of the mass of the oil phase, an aqueous pH of 6.5, an initiator dosage accounting for 0.03% of the total mass of the reaction system, a reaction temperature of 50 °C, and a reaction time of 4 h. The results of the study are shown in Figure 5. As can be seen from Figure 5, the apparent viscosity of polymer P(AM/AA/SSS/DMAAC-16) gel increases first and then decreases with the gradual increase in monomer dosage. This is because in a free radical polymerization reaction, a monomer molecule forms a free radical through an initiator or other external energy, which in turn triggers other monomeric molecules to form a new radical. If the monomer concentration is too low, the rate of free radical generation will be limited, which will affect the number of free radicals and the frequency of encounters between monomer molecules; the polymerization reaction rate will decrease, and the polymerization molecular chain will grow slowly, which will eventually lead to a smaller molecular weight of the product and a lower apparent viscosity of the polymer solution. With the gradual increase in the amount of monomer in a certain range, the effective content of monomer in the droplet increases, the encounter frequency between the initiator and the monomer molecule is guaranteed, the reaction rate of the polymerization reaction increases, the polymerization reaction chain grows steadily, and the apparent viscosity of the polymer solution gradually increases. When the monomer dosage accounted for 30% of the total mass of the reaction system, the apparent viscosity of the 1% polymer solution was observed to reach a maximum value of 109 mPa·s. If the amount of monomer continues to increase, the encounter frequency between the initiator and the monomer molecule will be too high, the polymerization reaction rate and reaction heat will rise sharply, and the internal heat of the reaction system will not easily be lost in a short time, which greatly increases the probability of chain termination in advance of the molecular chain of the polymerization product, which ultimately leads to the reduction in the molecular weight of the product and the apparent viscosity of the polymer solution. At the same time, excessive monomer dosage may also lead to side reactions such as cross-linking, branching, or shearing of the polymer chain, which will change the polymer structure and affect the properties and applications of the polymer. In summary, 30% is the optimal monomer dosage for the quaternary polymerization reaction system.

#### 3.1.5. Effect of Aqueous pH on Polymerization Reactions

**Figure 6:**
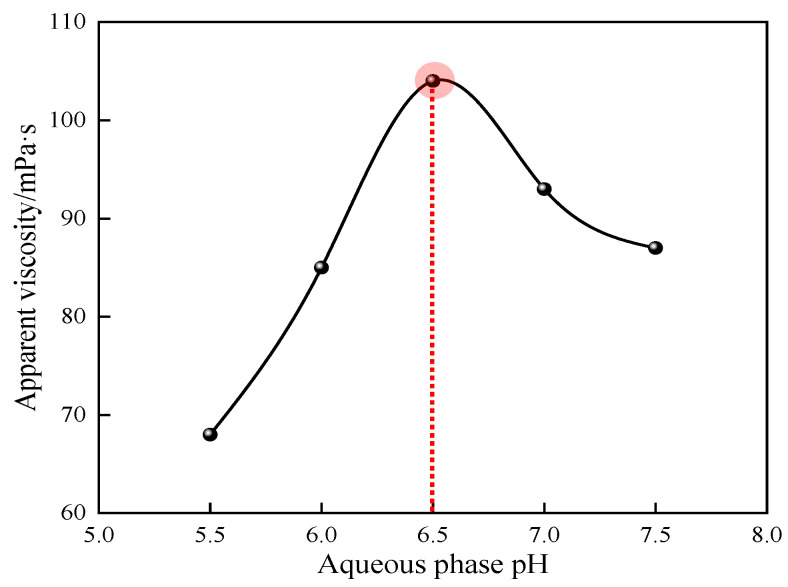
Effect of aqueous pH on the apparent viscosity of P(AM/SS/SSS/DMAAC-16) gel.

The effects of different aqueous pH ratios on the apparent viscosity of 1% quaternary polymer thickener solution were studied under the conditions of n(AM):n(AA):n(SSS):n(DMAAC-16) = 79.2:20:0.5:0.3, an oil–water volume ratio of 0.4, an emulsifier dosage that accounted for 7% of the oil phase mass, an monomer dosage that accounted for 30% of the total mass of the reaction system, an initiator dosage that accounted for 0.03% of the total mass of the reaction system, a reaction temperature of 50 °C, and a reaction time of 4 h. The results of the study are shown in Figure 6.

As can be seen from Figure 6, the apparent viscosity of polymer P(AM/AA/SSS/DMAAC-16) gel increases first and then decreases with the gradual increase in aqueous pH. This is because when the pH of the aqueous phase is too low, the activity of the initiator is low, the concentration of free radicals generated by its decomposition is not high, and the polymerization reaction rate is too slow. Moreover, AM is prone to immidylation in a strong acid environment, which makes it easy to form more branched chains during the polymerization reaction. These ultimately result in a lower molecular weight of the product and a less apparent viscosity of the polymer solution. With the increase in aqueous pH in a certain range, the activity of the initiator is gradually maximized, the polymerization reaction rate is increased, the polymerization reaction chain growth is carried out steadily, and the apparent viscosity of the polymer solution gradually increases. When the aqueous pH is 6.5, the maximum apparent viscosity of the 1% polymer solution is observed at 104 mPa·s. However, when the pH value of the aqueous phase is too high, the structure of the initiator will change, the activity will be reduced or even fail, and the polymerization reaction rate will slow down. In addition, AM is prone to hydrolysis to form NH_3_ in a strong alkaline environment, and the production rate of the chain transfer agent dextroamphetamine (NTP) is greatly increased, which greatly increases the probability of chain transfer and chain termination of the molecular chain of polymerization products. These ultimately result in a decrease in the apparent viscosity of the polymer solution. In summary, 6.5 is the optimal aqueous pH value of the quaternary polymerization reaction system.

#### 3.1.6. Effect of Initiator Dosage on Polymerization Reaction

**Figure 7:**
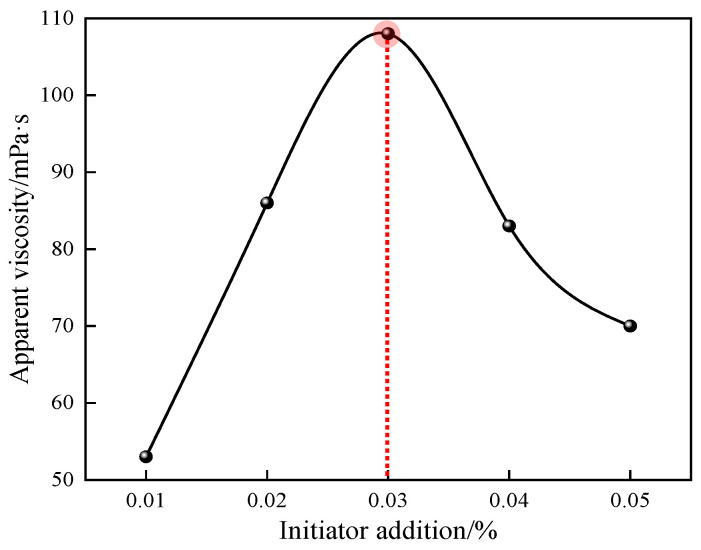
Effect of initiator dosage on apparent viscosity of P(AM/SS/SSS/DMAAC-16) gel.

The effects of different initiator dosage ratios on the apparent viscosity of 1% quaternary polymer thickener solution were studied under the conditions of n(AM):n(AA):n(SSS):n(DMAAC-16) = 79.2:20:0.5:0.3, an oil–water volume ratio of 0.4, an emulsifier dosage that accounted for 7% of the oil phase mass, a monomer dosage that accounted for 30% of the total mass of the reaction system, an aqueous phase pH of 6.5, a reaction temperature of 50 °C, and a reaction time of 4 h. The results of the study are shown in Figure 7.

As can be seen from Figure 7, the apparent viscosity of polymer P(AM/AA/SSS/DMAAC-16) gel increases first and then decreases with the increase in initiator dosage. This is because when the dosage of initiator is too low, the “cage reaction” is very likely to occur in the process of free radical polymerization; that is, the initiator molecule and its primary free radical decomposed by endothermic decomposition are in a state of being surrounded by solvent molecules for a long time and cannot react with the monomer molecule outside the cage, thus losing activity after the side reaction occurs in the cage, resulting in a decrease in the efficiency of the initiator, a slow polymerization reaction rate, a small molecular weight of the final product, and a low apparent viscosity of the polymer solution [13]. Within a certain range, when the amount of initiator is gradually increased, the rate of polymerization reaction gradually increases within a controllable range, the number of active free radicals and the collision frequency between monomer molecules are gradually maximized, the polymerization reaction chain grows steadily, and the apparent viscosity of the polymer solution gradually increases. When the initiator dosage was 0.03% of the total mass of the reaction system, the apparent viscosity of the 1% polymer solution was observed to be the largest, which was 108 mPa·s. However, when too much of the initiator is added, the initiator instantaneously decomposes more free radicals, the polymerization reaction rate is fast and the reaction easily gets out of control, the polymerization reaction heat in the system is difficult to dissipate, and the reaction is highly prone to overpolymerization, which ultimately leads to a decrease in the molecular weight of the product and the apparent viscosity of the polymer solution. In summary, 0.03% is the optimal initiator dosage for the quaternary polymerization reaction system.

#### 3.1.7. Effect of Reaction Temperature on Polymerization Reaction

**Figure 8:**
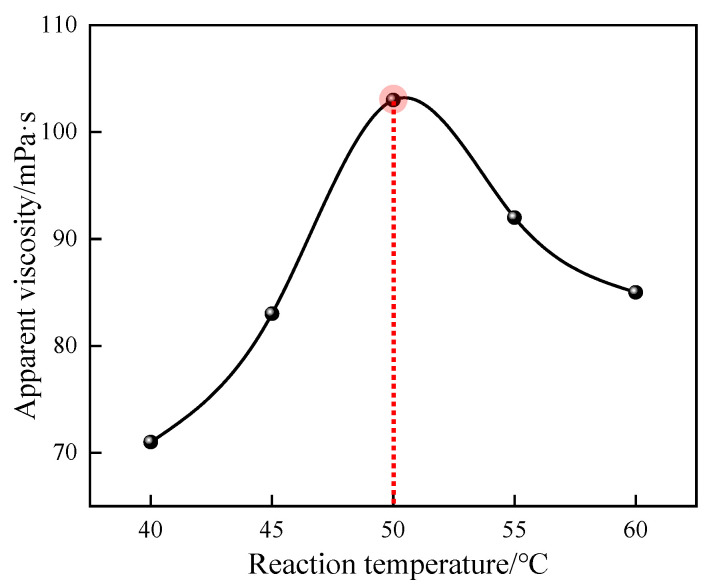
Effect of reaction temperature on apparent viscosity of P(AM/SS/SSS/DMAAC-16) gel.

The effects of different reaction temperatures on the apparent viscosity of 1% quaternary polymer thickener solution were studied under the conditions of n(AM):n(AA):n(SSS):n(DMAAC-16) = 79.2:20:0.5:0.3, an oil–water volume ratio of 0.4, an amount of emulsifier that accounted for 7% of the mass of the oil phase, an amount of monomer that accounted for 30% of the total mass of the reaction system, a pH of the aqueous phase of 6.5, an amount of initiator of 0.03%, and a reaction time of 4 h. The results of the study are shown in Figure 8.

As can be seen from Figure 8, the apparent viscosity of polymer P(AM/AA/SSS/DMAAC-16) gel increases first and then decreases with the gradual increase in reaction temperature. This is because when the reaction temperature is too low, the activity of the initiator will be affected, and the initiator will decompose slowly or even ineffectively start the polymerization reaction, thus making the polymerization reaction impossible. With the gradual increase in the reaction temperature in a certain range, the decomposition rate of the initiator gradually increases, the encounter frequency between the primary free radical and the monomer molecule gradually reaches the maximum, the polymerization reaction rate gradually increases within the controllable range, the polymerization reaction chain grows steadily, and the apparent viscosity of the polymer solution gradually increases. When the reaction temperature was increased to 50 °C, the apparent viscosity of the 1% polymer solution was the largest, which was 103 mPa·s. If the reaction temperature continues to increase, the decomposition rate of the initiator will be too fast, the collision between the primary free radical and the monomer molecule will be too intense, the polymerization reaction rate will be too fast and the reaction will easily get out of control, the polymerization reaction heat in the system will be difficult to dissipate, and the reaction will be prone to overpolymerization. In addition, the amide groups in AM are prone to degradation at high temperatures, which greatly increases the production rate of the chain transfer agent nzheenamine (NTP), which greatly increases the probability of chain transfer and chain termination of the polymerization product molecular chain [14]. In summary, 50 °C is the optimal reaction temperature for the quaternary polymerization reaction system.

#### 3.1.8. Effect of Reaction Time on the Polymerization Reaction

**Figure 9:**
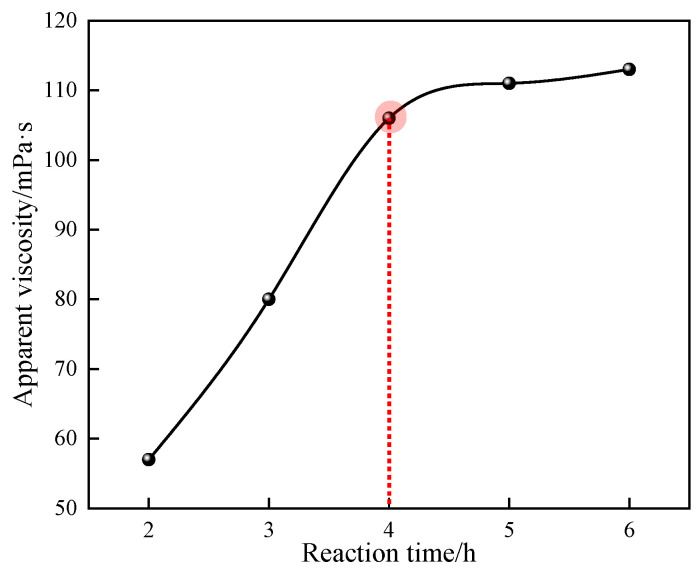
Effect of reaction time on the apparent viscosity of P(AM/SS/SSS/DMAAC-16) gel.

The effects of different reaction times on the apparent viscosity of 1% quaternary polymer thickener solution were studied under the conditions of n(AM):n(AA):n(SSS):n(DMAAC-16) = 79.2:20:0.5:0.3, an oil–water volume ratio of 0.4, an amount of emulsifier that accounted for 7% of the mass of the oil phase, an amount of monomer that accounted for 30% of the total mass of the reaction system, a pH of the aqueous phase of 6.5, a dosage of initiator that accounted for 0.03% of the total mass of the reaction system, and a reaction temperature of 50 °C. The results of the study are shown in Figure 9.

As can be seen from Figure 9, the apparent viscosity of polymer P(AM/AA/SSS/DMAAC-16) gel increases with the increase in reaction time, and gradually plateaus in the later stage of the reaction. This is because if the reaction time is too short, the polymerization reaction is incomplete, the polymer molecular chain is short, and the monomer conversion rate is low. With the gradual increase in the reaction time, the full reaction between the active free radical and the monomer molecule is gradually ensured, the integrity of the polymerization reaction is gradually improved, the polymerization reaction chain grows smoothly, and the apparent viscosity of the polymer solution gradually increases. When the reaction time was 4 h, the apparent viscosity of the 1% polymer solution was observed to be 106 mPa·s, and the apparent viscosity of the polymer solution continued to increase but did not increase much after 4 h. Combined with the time cost, in summary, 4 h is the optimal reaction time for the quaternary polymerization reaction system.

### 3.2. Performance Evaluation of Thickeners

#### 3.2.1. Evaluation of Thickening Performance

Conventional thickeners have to undergo an obvious swelling–dissolution process in the preparation process, and the dissolution time is long, and the construction efficiency of on-site application is low. The liquid polymer system prepared by the reversed-phase emulsion polymerization method has no swelling process in the solvent, so it has extremely high dissolution efficiency, which can meet the process requirements of continuous mixing on site. In this experiment, the apparent viscosity of the 1% polymer solution corresponding to the changed thickening time was used as the benchmark, and the relationship between the apparent viscosity of P(AM/AA/SSS/DMAAC-16) gel and the thickening time was investigated by using a six-speed rotational viscometer.

**Figure 10:**
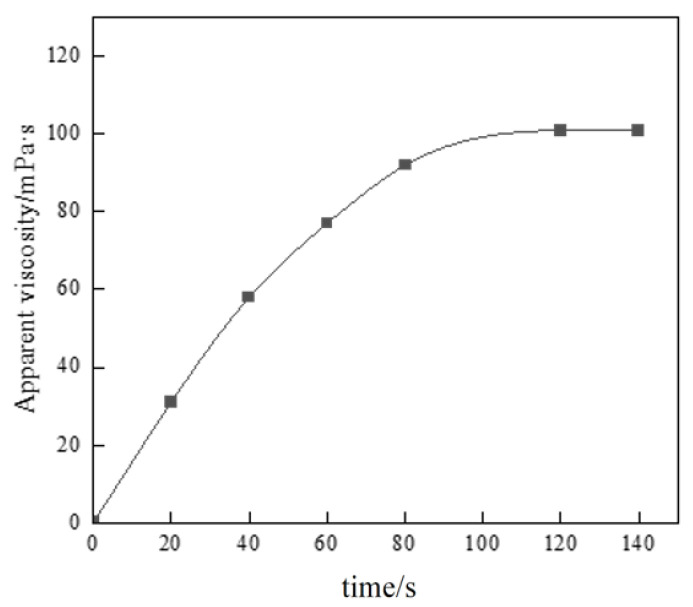
Thickening performance test of thickener.

As can be seen from Figure 10, the time of complete dissolution of thickener P (AM/AA/SSS/DMAAC-16) in clean water is less than or equal to 2 min. After 2 min, the polymer molecular chains were completely stretched, and the apparent viscosity reached the maximum value. When the dissolution time was 80 s, the apparent viscosity of the thickener solution gradually stabilized, and the apparent viscosity of the P(AM/AA/SSS/DMAAC-16) gel reached 92 mPa·s. From the above experimental results, it can be seen that the fracturing fluid thickener P(AM/AA/SSS/DMAAC-16) has good solubility in clean water.

#### 3.2.2. Evaluation of Temperature Resistance

**Figure 11:**
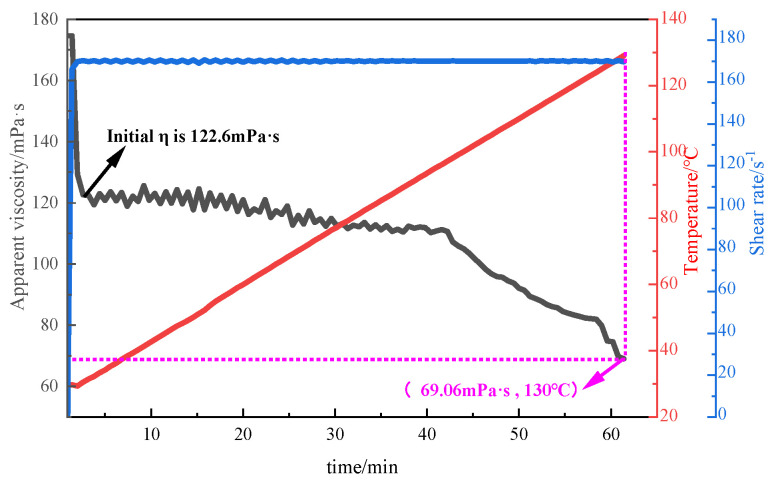
Temperature resistance curve of 1% P(AM/AA/SSS/DMAAC-16) thickener solution.

With the continuous exploitation of conventional oil and gas resources, oil and gas exploration and development has gradually expanded to unconventional oil and gas resources such as tight sandstone and deep low permeability reservoirs. The ground temperature gradient that fracturing fluids are subjected to during the fracturing process is also increasing. The increase in temperature has a serious impact on the effective viscosity of the polymer solution and the practical application effect. In this experiment, the temperature resistance of P(AM/AA/SSS/DMAAC-16) was tested by raising the test temperature to 130 °C with a heating rate of 0.05 °C/s using the MARS60 complex fluid flow tester. Firstly, the temperature resistance of the 1% thickener solution prepared with clean water was tested under the following conditions: the initial temperature was 30 °C, the heating rate was 0.05 °C/s, the termination temperature was 130 °C, and the shear rate was 170 s^−1^. The test results are shown in Figure 11. As can be seen from Figure 11, the apparent viscosity of the P(AM/AA/SSS/DMAAC-16) gel decreases with increasing temperature at a shear rate of 170 s^−1^. When the test temperature reaches 130 °C, the apparent viscosity of P(AM/AA/SSS/DMAAC-16) gel is 69.06 mPa·s, and the apparent viscosity loss rate is as high as 43.67%, and the apparent viscosity of the thickener solution meets the requirements of fracturing operation in the People’s Republic of China oil and gas industry standard [15] (SY/T 6376-2008) (fracturing fluid apparent viscosity ≥ 50 mPa·s). From the above experimental results, it can be seen that the fracturing fluid thickener P(AM/AA/SSS/DMAAC-16) has good temperature resistance in clean water.

#### 3.2.3. Evaluation of Temperature Resistance

**Figure 12:**
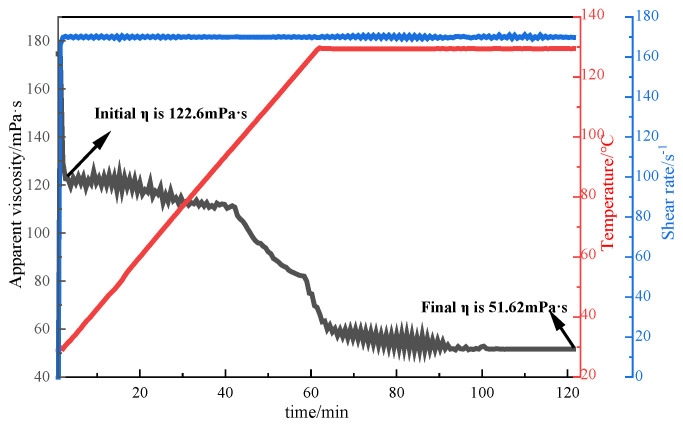
Shear resistance curve of 1% P(AM/AA/SSS/DMAAC-16) thickener solution.

Due to the high shear force of the conventional thickener solution, the molecular chain is easily damaged irreversibly, which seriously affects the apparent viscosity and practical application effect of the polymer solution. In order for the fracturing fluid to smoothly transport the proppant to the fracture during the fracturing process, it is necessary to ensure that the fracturing fluid can still provide sufficient apparent viscosity to carry sand under the action of high shear force. In this experiment, the MARS60 complex fluid flow tester was used to investigate the relationship between the apparent viscosity of P(AM/AA/SSS/DMAAC-16) gel and shear time. Firstly, the shear resistance of the 1% thickener solution prepared with clean water was tested under the following conditions: an initial temperature of 30 °C, a heating rate of 0.05 °C/s, a shear temperature of 130 °C, a shear rate of 170 s^−1^, and a shearing duration of 1 h. The test results are shown in Figure 12. As can be seen from Figure 12, the apparent viscosity of P(AM/AA/SSS/DMAAC-16) gel decreases with increasing shear time at a shear temperature of 130 °C. After continuous shearing at a shear rate of 170 s^−1^ for 1 h, the apparent viscosity of P(AM/AA/SSS/DMAAC-16) gel was 51.62 mPa·s, and the apparent viscosity loss rate was as high as 57.90%, which met the requirements of the industry standard (SY/T 6376-2008) for fracturing construction (apparent viscosity of fracturing fluid ≥ 50 mPa·s). From the above experimental results, it can be seen that the fracturing fluid thickener P (AM/AA/SSS/DMAAC-16) has a certain shear resistance in clean water.

#### 3.2.4. Evaluation of Salt Tolerance

In the process of fracturing, when the polymer solution comes into contact with a large number of different metal ions (such as Na^+^ and Ca^2+^) contained in the formation, the polymer molecular chain will shrink and curl to varying degrees due to salt sensitivity, which seriously affects the apparent viscosity and practical application effect of the polymer solution [16]. In this experiment, the apparent viscosity of the 1% polymer solution corresponding to the changed metal ion concentration was used as the benchmark, and the relationship curve between the metal ion concentration and the apparent viscosity of the P(AM/AA/SSS/DMAAC-16) gel was investigated by using a six-speed rotational viscometer.

**Figure 13:**
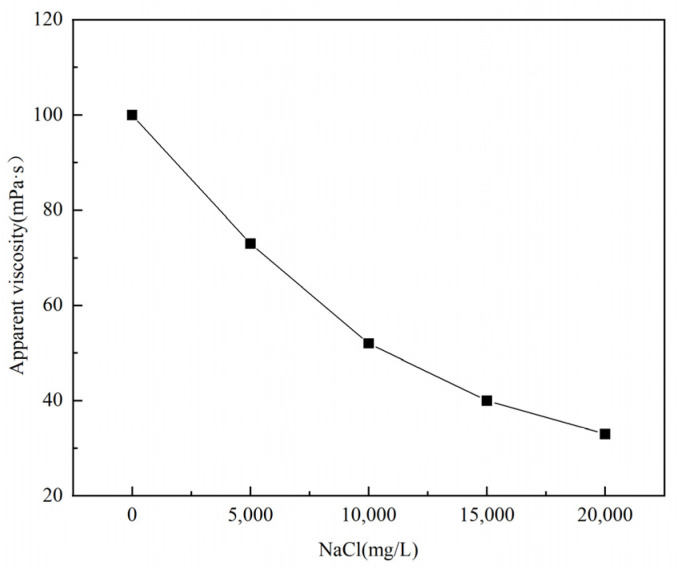
Effect of NaCl concentration on the apparent viscosity of thickener solution.

Firstly, the salt tolerance of the 1% thickener solution prepared from the NaCl solution with different gradient salinity was tested, and the test results are shown in Figure 13. As can be seen from Figure 13, the apparent viscosity of P(AM/AA/SSS/DMAAC-16) decreases sharply as the concentration of Na^+^ in the solution increases. When the Na^+^ concentration reaches 10,000 ppm, the viscosity loss of P(AM/AA/SSS/DMAAC-16) is huge, which is 51 mPa·s, which meets the requirements of the industry standard (SY/T 6376-2008) for fracturing operation (apparent viscosity of fracturing fluid ≥ 50 mPa·s). When the Na^+^ concentration continued to increase to 20,000 ppm, the apparent viscosity of P(AM/AA/SSS/DMAAC-16) was only 32 mPa·s, and the viscosity loss rate was as high as 68.32%. From the above experimental results, it can be seen that P(AM/AA/SSS/DMAAC-16) can tolerate a sodium salt concentration of up to 10,000 ppm, which is suitable for low-salinity formation.

**Figure 14:**
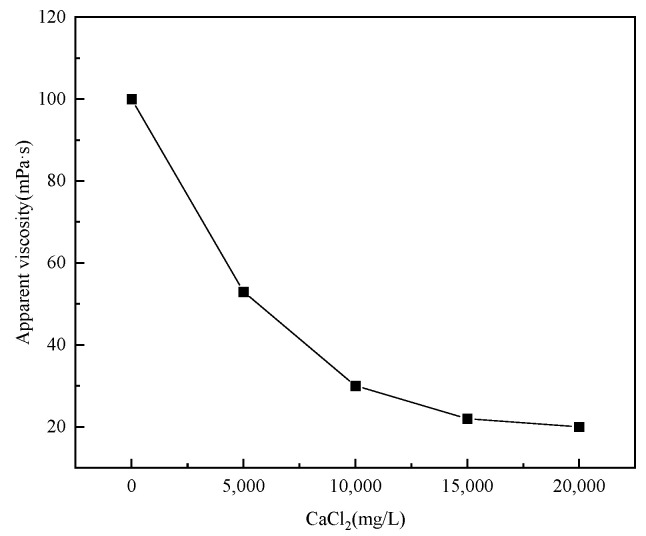
Effect of CaCl_2_ concentration on the apparent viscosity of thickener solution.

Then, the salt tolerance of the 1% thickener solution prepared by CaCl_2_ solution with different gradient salinity was tested, and the test results are shown in Figure 14. As can be seen from Figure 14, the apparent viscosity of P(AM/AA/SSS/DMAAC-16) gel decreases sharply as the concentration of Ca^2+^ in the solution increases. When the Ca^2+^ concentration reaches 10,000 ppm, the viscosity loss of P(AM/AA/SSS/DMAAC-16) is huge, and only 30 mPa·s remains, which does not meet the requirements of the industry standard (SY/T 6376-2008) for fracturing operation at all (apparent viscosity of fracturing fluid ≥ 50 mPa·s). The apparent viscosity of P(AM/AA/SSS/DMAAC-16) is less than 22 mPa·s, and the viscosity loss rate is not less than 77.55%. From the above experimental results, it can be seen that the resistance of P(AM/AA/SSS/DMAAC-16) to calcium salt is much weaker than that of sodium salt, and the ability of high-valent metal ions to destroy the molecular chain structure of polymers is stronger than that of low-valent metal ions.

#### 3.2.5. Evaluation of Viscoelastic Properties

**Figure 15:**
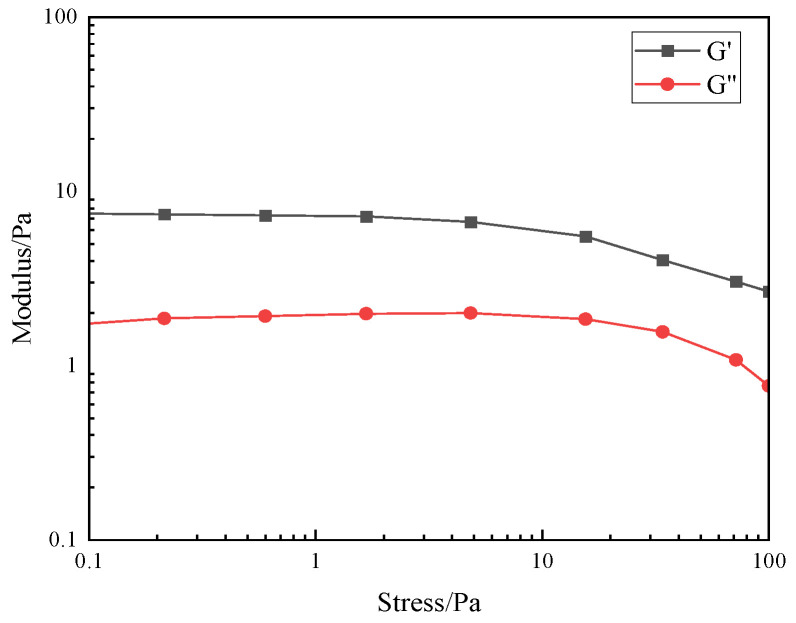
Stress scan of 1% thickener P(AM/AA/SSS/DMAAC-16) gel.

**Figure 16:**
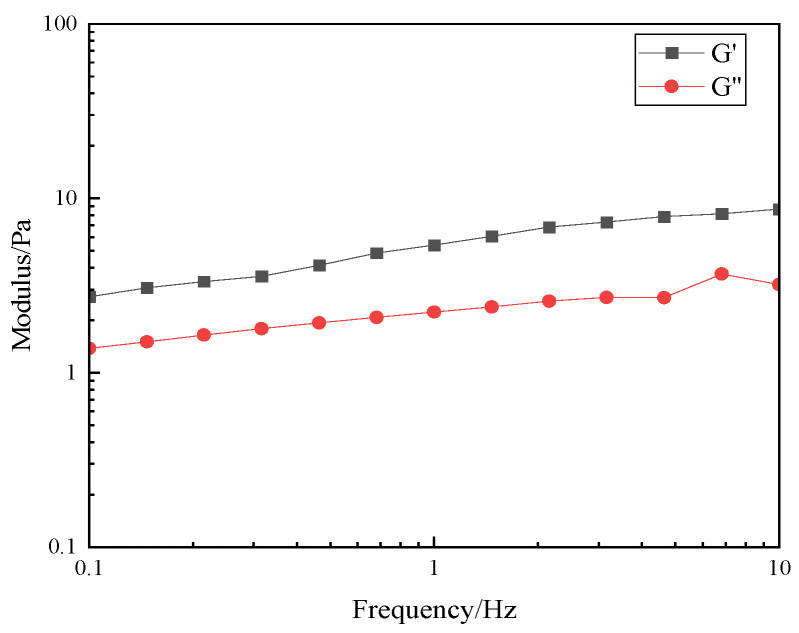
Frequency scan of 1% thickener P(AM/AA/SSS/DMAAC-16) gel.

The viscoelasticity of fracturing fluids is the essence that affects their sand-carrying properties [17]. Fracturing fluids with appropriate viscoelastic properties can not only coat the sand particles better and form a more stable liquid bridge in the fractures, thereby improving the suspension capacity of the sand particles and maintaining the efficiency of pressure transmission, but also increase the interaction force between the sand particles and the liquid and reduce the risk of sand deposition and blockage. In this experiment, the linear viscoelastic interval of the thickener solution was obtained by using the MARS60 complex fluid flow tester, and the relationship between the elastic modulus (G′) and the viscous modulus (G″) with frequency was investigated, and then the viscoelastic performance of the thickener solution was evaluated. As can be seen from Figure 15 and Figure 16, the elastic modulus (G′) of the thickener solution is always greater than that of the viscous modulus (G″), which indicates that the P(AM/AA/SSS/DMAAC-16) thickener solution is mainly elastic, which indirectly indicates that the P(AM/AA/SSSS/DMAAC-16) thickener solution has a strong sand suspension ability.

#### 3.2.6. Evaluation of Suspended Sand Performance

In the process of fracturing construction, the sand suspension capacity of the fracturing fluid directly affects the sand removal speed of the proppant, if the sand suspension capacity of the fracturing system is poor, the proppant can easily demand work too quickly and then settles at the bottom of the wellbore, resulting in accidents such as sand plugging and sand jamming at the bottom of the well. In this experiment, 20/40 ceramsite was used as the proppant to observe the sand-carrying state of 20%, 30%, 50%, and 100% sand compared to 1% thickener solution within 30 min, and the sand-carrying capacity of the polymer solution was evaluated by measuring the sedimentation rate of the proppant [18,19]. Data on the sand-carrying capacity of the solution are shown in Table 2

**Figure 17:**
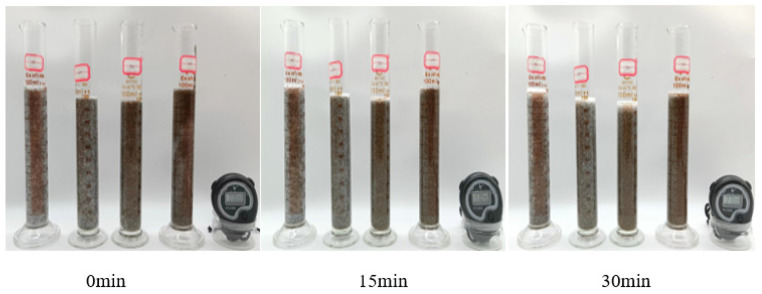
Sand-carrying state diagram of 1% P(AM/AA/SSS/DMAAC-16) thickener solution—room temperature.

**Figure 18:**
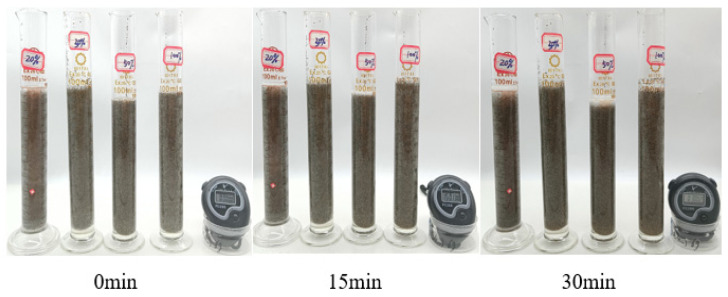
Sand-carrying state of 1% P(AM/AA/SSS/DMAAC-16) thickener solution at −90 °C.

From Figure 17 and Figure 18, it can be seen that when the sand-carrying capacity of the thickener solution was tested at room temperature and 90 °C, the 1% thickener solution at 20%, 30%, 50%, and 100% sand ratios did not settle significantly within 30 min. As can be seen from Table 3 and Table 4, when the sand ratio is 100%, the settling rate of proppant in P(AM/AA/SSS/DMAAC-16) thickener solution is 0.062 cm/min at room temperature. At 90 °C, the sedimentation rate of proppant in P(AM/AA/SSS/DMAAC-16) thickener solution was 0.064 cm/min, and it can be seen from the above experimental results that the thickener P(AM/AA/SSS/DMAAC-16) had good sand-carrying performance.

#### 3.2.7. Evaluation of Gel Breaking Performance

In the process of fracturing, the fracturing fluid carries the proppant into the fracture by virtue of its high viscosity to achieve the purpose of fracture creation and diversion. When the fracturing transformation is completed, in order to reduce the damage to the formation, it is necessary to break the gel and flowback of the fracturing fluid in the formation. The smaller the surface tension and interfacial tension of the broken gel, the lower the residue content and the more conducive it is to reducing the capillary resistance and the damage to the formation, as well as increasing the flowback capacity of the broken gel. In this experiment, the surface tension of the thickener gel breaking solution was measured by a surface tension meter (QBZY series), a rotary drop interfacial tensiometer (SITE 100), and a low-speed centrifuge (SC-03). Combined with the above test indicators, the performance evaluation of the thickener gel breaking was carried out.

**Figure 19:**
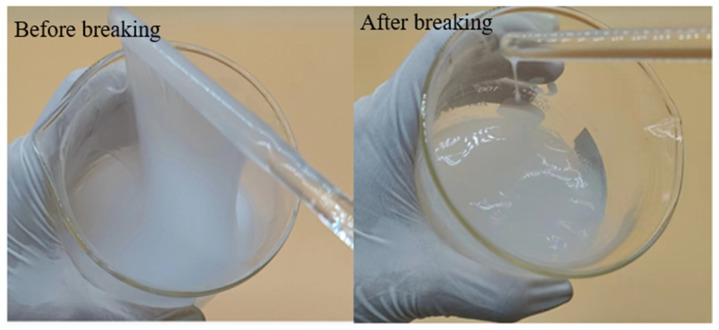
Diagram of 1% P(AM/AA/SSS/DMAAC-16) thickener solution before and after breaking at 90 °C.

As can be seen from Table 3, the thickener P(AM/AA/SSS/DMAAC-16) can completely break the gel within 4 h at 90 °C and 0.04% ammonium persulfate. After the gel breaking treatment, the apparent viscosity value, surface tension value, interfacial tension value and residue content value of the gel breaking solution meet the general conditions requirements of the industry standard (SY/T 6376-2008) for fracturing fluid technology (the apparent viscosity of the gel breaking solution ≤ 5 mPa·s, the surface tension ≤ 28 mN/m, the interfacial tension between the gel breaking solution and kerosene ≤ 2 mN/m, and the residue content of the gel breaking solution ≤ 600 mg/L). From the above experimental results, it can be seen that the thickener P(AM/AA/SSS/DMAAC-16) has good gel breaking performance. The effect of the broken gel solution is shown in Figure 19

#### 3.2.8. Core Damage Performance Evaluation

**Figure 20:**
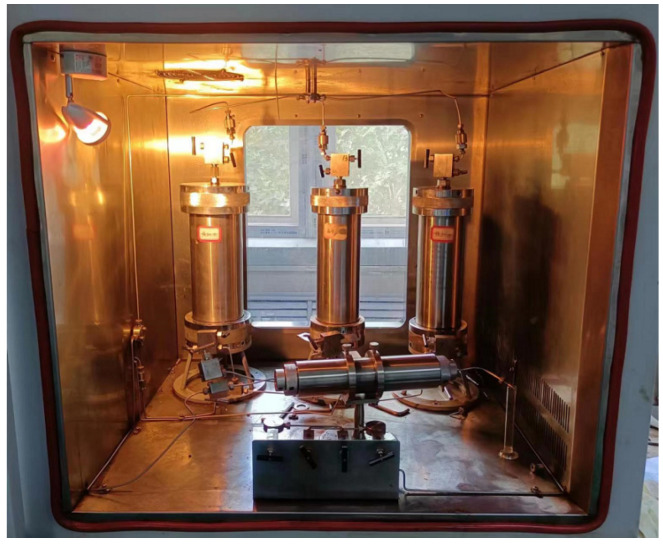
Evaluation device for water shut-off profile control.

In the process of fracturing transformation, the degree of damage to the rock reservoir by fracturing fluid is the key factor affecting the effect of fracturing operation. In order to simulate the process of water injection profile control in actual reservoir development and evaluate the possible damage of the core after water injection profile control operation, according to the industry standard (SY/T 5107-2016), the influence of 1% fracturing fluid thickener solution on core permeability was investigated by using a water shut-off profile control evaluation device (as shown in Figure 20). Combined with the core damage rate, the damage ability of fracturing fluid to the formation was evaluated. In this experiment, formation water was selected as the flow medium, the artificial core was used as the test core, and the test temperature was 60 °C. The experimental procedure is as follows: Determination of core permeability K_1_ before damage: The saturated artificial core is put into the core holder of the water shut-off profile control evaluation device, and the formation water is injected into the core from one end of the clamp for displacement operation (injection direction: consistent with the flow direction of reservoir fluid), the formation water flow rate is controlled at 3 mL/min, and the stable displacement for 1 h after the formation water solution flows out to obtain a stable pressure difference P_1_. Core damage process: The gel breaking fluid of the fracturing fluid is placed into a high-pressure vessel, and the pressurization operation is carried out with the help of a pressure source, so that the gel breaking solution enters the core from one end of the gripper (injection direction: opposite to the flow direction of the reservoir fluid), and the gel breaking solution stays in the core for 2 h. Determination of core permeability K_2_ after damage: The formation water is injected into the core from one end of the gripper for displacement operation (injection direction: consistent with the flow direction of reservoir fluid), the formation water flow rate is controlled at 3 mL/min, and the stable displacement is obtained after the formation water solution flows out for 1 h to obtain a stable pressure difference P_2_. The core permeability formula is as follows (Equation (2)):

(2) $$K=\frac{Q\mu L}{∆\rho A}\times 10^{-1}$$

K—liquid permeability, μm^2^; Q—flow rate, cm^3^/s; μ—viscosity of the gel breaking solution at the test temperature, mPa·s; L—length of the core, cm; differential pressure through the core, MPa; A—cross-sectional area of the core, cm^2^.

The matrix permeability damage rate formula is as follows (Equation (3)):

(3) $$\eta_{d}=\frac{K_{1}-K_{2}}{K_{2}}\times 100\%$$

η—permeability damage rate, %; K_1_—core permeability before damage, μm^2^; K_2_—core permeability after damage, μm^2^.

From Equations (2) and (3), the damage rate of the fractured fluid to the core permeability can be calculated. The experimental results are shown in Table 4.

It can be seen from Table 4 that at 60 °C, the damage rate of P(AM/AA/SSS/DMAAC-16) gel breaking solution to the core is 28.77%, which is 30% lower than the limit value of the general conditions of fracturing fluid technology required by the industry standard (SY/T 6376-2008). From the above experimental results, it can be seen that the thickener P(AM/AA/SSS/DMAAC-16) has low damage to rock reservoirs and has little impact on the environment in the actual application process.

#### 3.2.9. Reuse of Fracturing Fluid Flowback Fluid

In order to improve the effective utilization of resources, many scholars at home and abroad have proposed the technology of recycling and reusing the fracturing fluid flowback fluid [20,21,22,23,24,25,26,27,28]. Due to the high concentration of salts and a large number of metal ions such as Na^+^, Mg^2+^, Ca^2+^, etc., the molecular chains of polymers are prone to curling, which in turn affects the performance of repeatedly formulated fracturing fluids. In view of the complex characteristics of flowback fluid quality, it is necessary to investigate the properties of the thickener solution prepared by fracturing fluid flowback.

##### Evaluation of Thickening Performance

In this experiment, the fracturing fluid thickener was prepared into a 2% thickener solution with fracturing flowback fluid, and then the six-speed rotational viscometer was used to explore the relationship between the apparent viscosity of P(AM/AA/SSS/DMAAC-16) gel with thickening time according to the operation method in 2.3.

**Figure 21:**
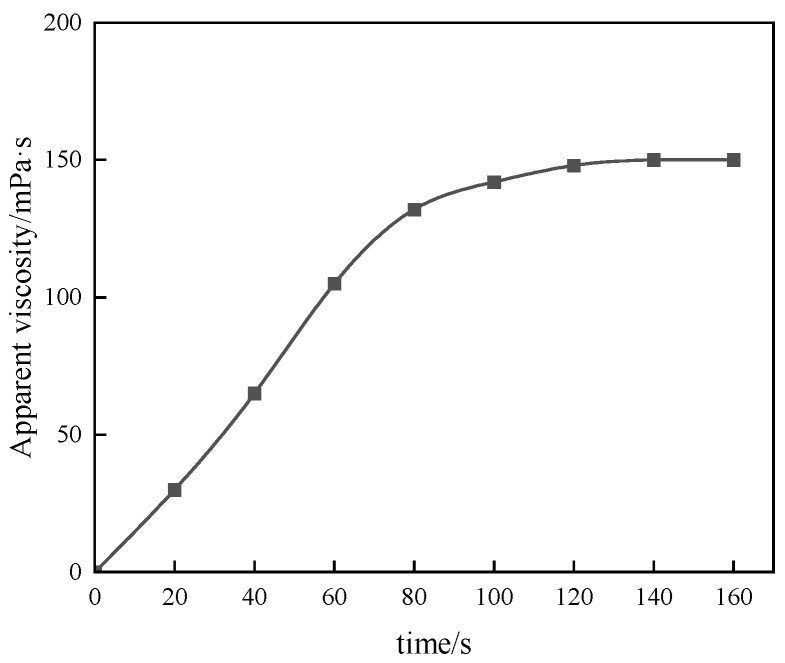
Test of thickening performance of thickener in fracturing fluid flowback fluid.

As can be seen from Figure 21, the complete dissolution time of P(AM/AA/SSS/DMAAC-16) in the fracturing flowback fluid is less than 3 min. After 3 min, the polymer molecular chains were completely stretched, and the apparent viscosity reached the maximum value. It can be seen from the above experimental results that the apparent viscosity of P(AM/AA/SSS/DMAAC-16) thickener solution has gradually stabilized when the dissolution time is 2 min, and the apparent viscosity of P(AM/AA/SSS/DMAAC-16) gel can reach 148 mPa·s. From the above experimental results, it can be seen that the thickener P(AM/AA/SSS/DMAAC-16) has good solubility in the fracturing fluid flowback fluid.

##### Evaluation of Temperature Resistance

**Figure 22:**
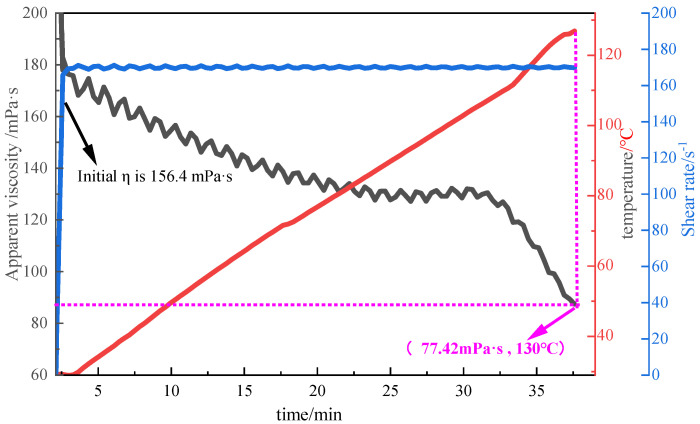
Temperature resistance curve of 2% P(AM/AA/SSS/DMAAC-16) thickener flowback solution.

In this experiment, the fracturing fluid thickener was prepared into a 2% thickener solution with fracturing flowback fluid, and then the temperature resistance of P(AM/AA/SSS/DMAAC-16) was tested by MARS60 complex fluid flow tester. The test conditions were as follows: an initial temperature of 30 °C, a heating rate of 0.05 °C/s, a termination temperature of 130 °C, and a shear rate of 170 s^−1^. The test results are shown in Figure 22.

As can be seen in Figure 22, the apparent viscosity of P(AM/AA/SSS/DMAAC-16) gel decreases with increasing temperature at a shear rate of 170 s^−1^. When the test temperature reaches 130 °C, the apparent viscosity of P(AM/AA/SSS/DMAAC-16) thickener solution is 77.42 mPa·s, and the apparent viscosity loss rate is as high as 50.50%. The apparent viscosity of the thickener solution meets the requirements of fracturing in the People’s Republic of China oil and gas industry standard (SY/T 6376-2008) (fracturing fluid apparent viscosity ≥ 50 mPa·s). From the above experimental results, it can be seen that the fracturing fluid thickener P(AM/AA/SSS/DMAAC-16) has good temperature resistance in the fracturing fluid flowback fluid.

##### Evaluation of Shear Resistance

**Figure 23:**
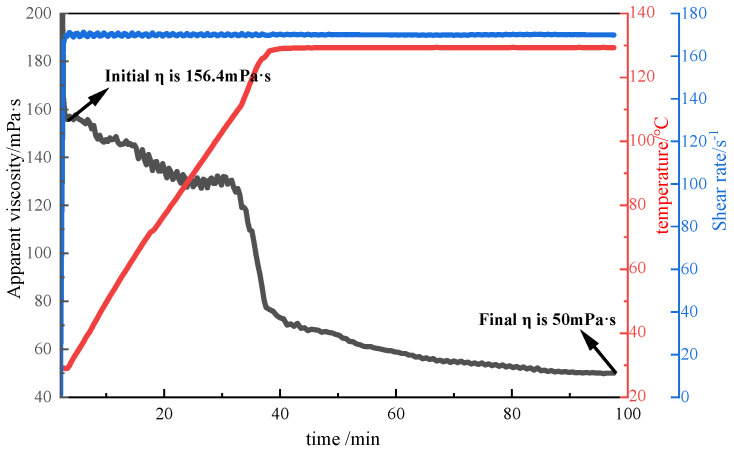
Shear resistance curve of 2% P(AM/AA/SSS/DMAAC-16) thickener flowback fluid.

In this experiment, the fracturing fluid thickener was prepared into a 2% thickener solution with fracturing flowback fluid, and then the shear resistance of P (AM/AA/SSS/DMAAC-16) was tested by MARS60 complex fluid flow tester. The test conditions were as follows: an initial temperature of 30 °C, a heating rate of 0.05 °C/s, a shear temperature of 130 °C, a shear rate of 170 s^−1^, and a shearing duration of 1 h. The test results are shown in Figure 23. As can be seen from Figure 23, the apparent viscosity of the P(AM/AA/SSS/DMAAC-16) thickener solution prepared with fracturing fluid flowback fluid as solvent decreases with the increase in shear time at 130 °C. After continuous shearing at a shear rate of 170 s^−1^ for 1 h, the apparent viscosity of P(AM/AA/SSS/DMAAC-16) gel is 50 mPa·s, and the apparent viscosity loss rate is as high as 68.03%, which fully meets the requirements of the industry standard (SY/T 6376-2008) for fracturing fluid construction; that is, the apparent viscosity of fracturing fluid is not less than 50 mPa·s. From the above experimental results, it can be seen that the thickener P(AM/AA/SSS/DMAAC-16) has a certain shear resistance in the fracturing fluid flowback.

##### Evaluation of Suspended Sand Performance

**Figure 24:**
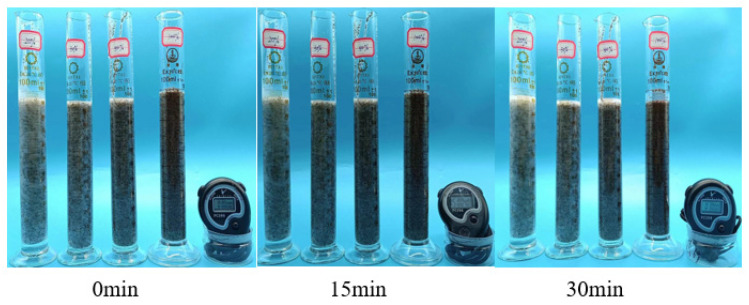
Diagram of the sand-carrying state of 1% P(AM/AA/SSS/DMAAC-16) thickener flowback liquid.

In this experiment, the fracturing fluid thickener was prepared into a 2% thickener solution with fracturing flowback fluid, and then the ceramsite with a mesh number of 20/40 was selected as the proppant, and the sand-carrying state of the thickener solution under the sand ratios of 20%, 30%, 50%, and 100% within 30 min was observed by the static sand-carrying method, and the sand-carrying capacity of the polymer solution was evaluated by measuring the sedimentation rate of the proppant. As can be seen from Figure 24, when the fracturing fluid thickener is added at 2%, the sand ratio of 20%, 30%, 50%, and 100% proppant is added, and the proppant does not settle significantly within 30 min. As can be seen from Table 4, Table 5 and Table 6, when the sand ratio is 100%, the settling rate of proppant in P(AM/AA/SSS/DMAAC-16) thickener solution is only 0.058 cm/min at room temperature.

##### Evaluation of Gel Breaking Performance

In this experiment, 2% fracturing fluid thickener was prepared into a thickener solution with fracturing flowback fluid, and then the surface tension, interfacial tension and residue content of the thickener solution were tested by a surface tension instrument (QBZY series), a rotary drop interfacial tension meter (SITE 100), and a low-speed centrifuge (SC-03). Combined with the above test indexes, the gel breaking performance of the thickener solution was comprehensively evaluated.

It can be seen from Table 6 that when the fracturing fluid thickener P(AM/AA/SSS/DMAAC-16) is added to 2%, the thickener solution can be completely broken within 4 h at 90 °C and 0.04% ammonium persulfate. Moreover, the apparent viscosity, surface tension, interfacial tension, and residue content values of the two gel breaking fluids meet the general conditions of fracturing fluid technology required by the industry standard (SY/T 6376-2008). From the above experimental results, it can be seen that the thickener P(AM/AA/SSS/DMAAC-16) has good gel breaking performance in the fracturing fluid flowback fluid and has little impact on the environment in the actual application process.

##### Molecular Weight of the Polymer

The characteristic viscosity number and relative molecular weight of fracturing fluid thickener P(AM/AA/SSS/DMAAC-16) were analyzed by using an automatic kinematic viscosity tester (SYD-265H), and the results are shown in Table 7.

### 3.3. Experimental Characterization Analysis

#### 3.3.1. X-Ray Diffraction Analysis (XRD)

**Figure 25:**
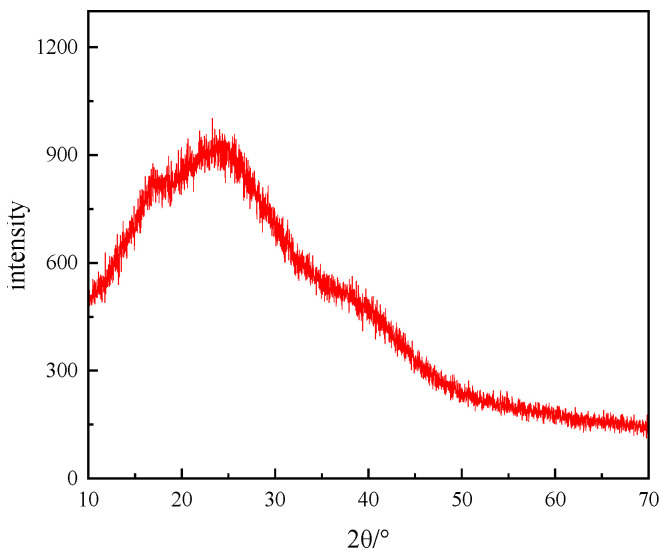
XRD spectra of thickener P(AM/AA/SSS/DMAAC-16).

The crystalline properties of polymers were investigated using a D8 ADVAHCL X-ray polycrystal diffractometer. As can be seen in Figure 25, there is a broad–diffuse peak in the spectrum. This result is due to the fact that in the polymerization reaction, monomeric molecules will be randomly added to the molecular chain, and then there are a large number of disordered structures in the polymer molecular chain, such as randomly arranged chain segments, irregular branches or side groups, etc., which leads to the inability of polymer molecules to form a regular crystal structure. In addition, the intertwining of polymer chains can also produce an amorphous structure of a three-dimensional network. The XRD diffraction structure indicates that there is no crystal structure in the polymer, which is consistent with the product characteristics of the target.

#### 3.3.2. Infrared Spectroscopy (FTIR)

**Figure 26:**
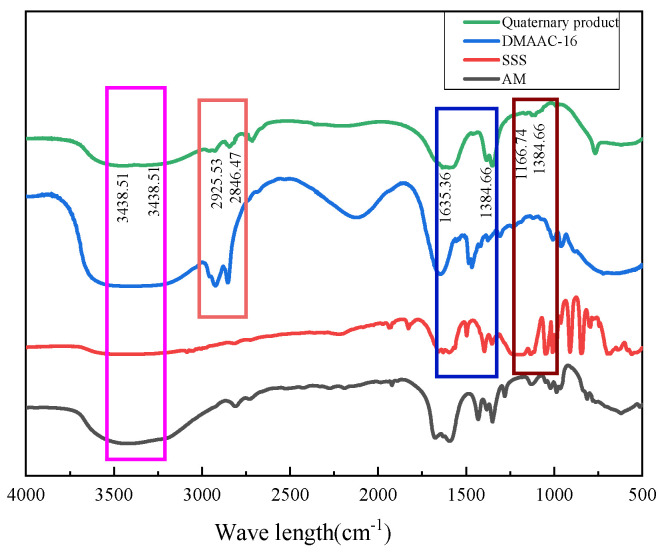
Infrared spectra of thickener P(AM/AA/SSS/DMAAC-16).

The type and molecular structure of polymers were investigated using the Nicolet 5700 infrared spectrometer [29,30,31,32]. As can be seen from Figure 26, 3438.51 cm^−1^ and 1635.36 cm^−1^ are the expansion and contraction vibration peaks of N-H and C=O in acrylamide (AM), respectively, indicating the existence of polyacrylamide structure in the polymer. Furthermore, 2925.53 cm^−1^ and 2846.47 cm^−1^ are the characteristic absorption peaks of the methyl and methylene groups on the hydrophobic long chain, respectively, and 3315.31 cm^−1^ is the absorption peak of the quaternary ammonium salt-N^+^(CH_3_)_2_-R, which can prove the existence of cetyl dimethylallyl ammonium chloride (DMAAC-16) in the polymer. At the same time, 1384.66 cm^−1^ is the characteristic peak of benzene ring, and 1106.96 cm^−1^ is the symmetrical stretching vibration peak of S=O bond in sulfonic acid group, which proves that sodium *p*-styrene sulfonate (SSS) has been successfully introduced into the polymer.

#### 3.3.3. Thermogravimetric Analysis (TGA)

**Figure 27:**
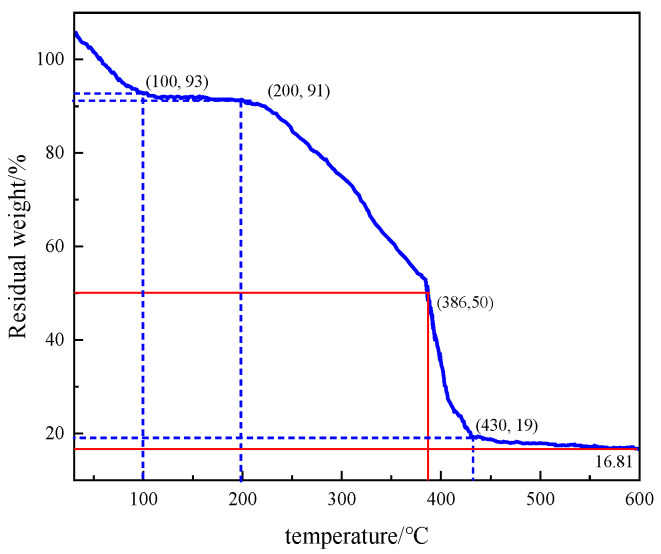
Thermogravimetric analysis of the thickener P(AM/AA/SSS/DMAAC-16).

The thermal stability of polymers was investigated using a TGA thermogravimetric analyzer. As can be seen from Figure 27, the thermal decomposition process of quaternary polymers is mainly divided into four parts: When the temperature range is 30–100 °C, the TG curve can analyze the thermal weight loss at this stage to be 7%. Because the polymer has strong hydrophilicity under the joint action of 2-acrylamide-2-methylpropanesulfonic acid and the sulfonic group of sodium p-styrene sulfonate and the carboxylic acid group of acrylic acid, the weight loss of the polymer at this stage is mainly due to its adsorption of water and free water. In the temperature range of 100–210 °C, the weight loss of the polymer is 2% due to the decomposition of some residual monomers that do not participate in the polymerization reaction of the reversed-phase emulsion. In the temperature range of 210–430 °C, the thermal weight loss is 72%, and the weight loss of the polymer at this stage mainly comes from the decomposition of some functional groups on the polymer molecular chain, including the cleavage of amide groups and the thermal decomposition of sulfonate, allyl, and benzene rings. In the temperature range of 430–600 °C, the thermal weight loss at this stage is 2.19% due to the decomposition of the polymer backbone, and the final residual weight of the polymer is 16.81%. From the above analysis, it can be seen that the polymer P(AM/AA/SSS/DMAAC-16) has strong thermal stability between 100 and 200 °C and can be used at higher temperatures.

#### 3.3.4. NMR Analysis

As shown in Figure S1 in the Supplementary Materials, the O-H single peak is at δ = 5.35, the N-H peak is at δ = 3.65, the H signal peak on the C connected to the carbonyl group is at δ = 1.26, and the hydrogen signal peak of the methylene group is at δ = 0.88. As shown in Figure S2, there is a strong signal at the chemical shift of δ = 77.16, which is the solvent peak of deuterated chloroform, the signal peak group of carbonyl carbon at δ = ~170, the signal peak group of C on the benzene ring at δ = ~128, the signal peak of C connected to the carbonyl group at δ = 29.55, and the signal peak of methylene C at δ = 22.53. These characterizations demonstrate the chemical composition of the synthesized polymer.

## 4. Conclusions

In order to develop an instant and rapidly thickening fracturing fluid thickener in water, AM, AA, SSS, and DMAAC-16 were used as functional monomers, and the emulsion-type fracturing fluid thickener P(AM/AA/SSS/DMAAC-16) was prepared by reversed-phase emulsion polymerization. The optimal synthesis conditions of P(AM/AA/SSS/DMAAC-16) were as follows: the oil–water volume ratio was 0.4, the emulsifier dosage was 7% of the oil phase mass, the initiator dosage was 0.03% of the total mass of the reaction system, the reaction time was 4 h, the reaction temperature was 50 °C, the aqueous phase pH was 6.5, and the monomer dosage was 30% of the total mass of the reaction system (monomer molar ratio n(AM):n(AA):n(SSS):n(DMAAC-16) = 79.2:20:0.5:0.3). Through a series of performance evaluation experiments of the thickener, the results show that P(AM/AA/SSS/DMAAC-16) can be completely dissolved in clean water within 2 min. Under the experimental conditions of 170 s^−1^ and 130 °C, the apparent viscosity of the solution remained above 50 mPa·s after shearing for 1 h; that is, P(AM/AA/SSS/DMAAC-16) had good temperature and shear resistance. Moreover, the fracturing fluid system prepared by P(AM/AA/SSS/DMAAC-16) also has the advantages of good sand suspension capacity, low content of gel breaking liquid residue, small damage to the reservoir, and reuse of fracturing fluid flowback fluid, which can meet the needs of oilfields.

## Figures and Tables

**Table 1:** Effect of monomer molar ratio on apparent viscosity of P(AM/SS/SSS/DMAAC-16) gel.

Serial Number	n(AM):n(AA):n(SSS):n(DMAAC-16)	Apparent Viscosity (mPa·s)
1	83.2:16:0.4:0.4	82
2	81.2:18:0.4:0.4	86
3	79.2:20:0.4:0.4	92
4	77.2:22:0.4:0.4	72
5	79.2:20:0.3:0.5	68
6	79.2:20:0.2:0.6	53
7	79.2:20:0.5:0.3	109
8	79.2:20:0.6:0.2	86

**Table 2:** Evaluation of the sand-carrying capacity of the thickener solution.

Aggregation System	Ratio of Sand and Liquids/%	Sedimentation Rate (cm/min)	
		Room Temperature 90 °C	
P(AM/AA/SSS/DMAAC-16)	20	0.023	0.033
	30	0.031	0.040
	50	0.042	0.053
	100	0.062	0.064

**Table 3:** Evaluation of gel breaking performance of thickener solution.

Type of Solution	Gel Breaking Time (h)	Apparent Viscosity of Gel Breaking Solution (mPa·s)	The Surface Tension of the Gel Breaking Solution (mN/m)	Gel Breaking Interface Tension (mN/m)	Residue Content (mg/L)
1% Thickener gel (cm^3^/s)	4	4.515	27.3	1.689	123

**Table 4:** Test data of fracture breaking fluid on core permeability damage rate.

Test Temperature (°C)	Flowing Medium	Core Damage Specifications		Core Permeability (μm^2^)		Damage Rate (%)
		Length (cm^3^/s)	Diameter (cm^3^/s)	Before Damage K_1_	After Damage K_2_	
60	Gel breaking	4.876	2.53	0.073	0.052	28.77

**Table 5:** Evaluation of the sand-carrying capacity of the thickener solution and flowback solution.

Aggregation System	Ratio of Sand and Liquids/%	Sedimentation Velocity (cm/min)
P(AM/AA/SSS/DMAAC-16)	20	0.018
	30	0.023
	50	0.034
	100	0.058

**Table 6:** Evaluation of gel breaking performance of thickener solution flowback liquid.

Type of Solution	Gel Breaking Time (h)	Apparent Viscosity of Gel Breaking Solution (mPa·s)	The Surface Tension of the Gel Breaking Solution (mN/m)	Gel Breaking Interface Tension (mN/m)	Residue Content (mg/L)
1% thickener solution—flowback solution	4	4.768	28.2	1.739	198

**Table 7:** Relative molecular mass test of thickener P (AM/SS/SSS/DMAAC-16).

Polymers	t_0_ (s)	t (s)	η_r_	[η] (dl·g^−1^)	M
P(AM/AA/SSS/DMAAC-16)	117	312	2.67	15.04	8,773,106

## Data Availability

The original contributions presented in the study are included in the article/Supplementary Materials, further inquiries can be directed to the corresponding authors.

## Supplementary Materials

The following supporting information can be downloaded at: https://www.mdpi.com/article/10.3390/polym17020217/s1, Figure S1. H-NMR spectrum of P(AM/AA/SSS/DMAAC-16); Figure S2. C-NMR spectrum of P(AM/AA/SSS/DMAAC-16).
