# Supplementary material for: Synthesis of P(AM/AA/SSS/DMAAC-16) and Studying Its Performance as a Fracturing Thickener in Oilfields
Source: Polymers (Basel). 2025 Jan 16;17(2):217. doi: 10.3390/polym17020217 (PMC11768575; doi:10.3390/polym17020217)
Supplement: Supplementary file 1 [file polymers-17-00217-s001.zip › polymers-3200375-supplementary.pdf]

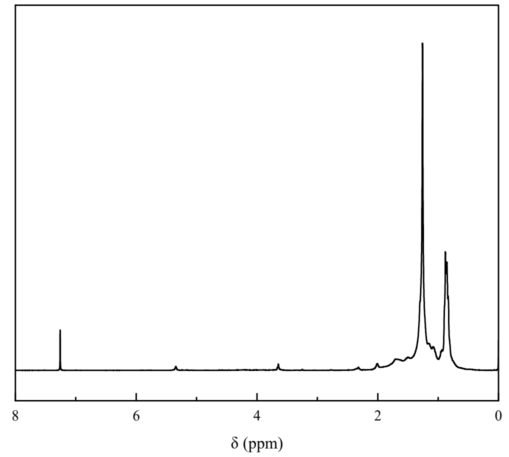

Figure 1(S) H-NMR spectrum of P(AM/AA/SSS/DMAAC-16)

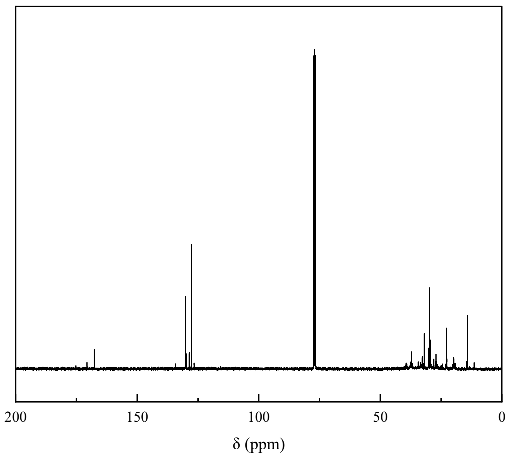

Figure 2(S) C-NMR spectrum of P(AM/AA/SSS/DMAAC-16)
